# Dietary intake and stress fractures among elite male combat recruits

**DOI:** 10.1186/1550-2783-9-6

**Published:** 2012-03-13

**Authors:** Daniel S Moran, Yuval Heled, Yael Arbel, Eran Israeli, Aharon S Finestone, Rachel K Evans, Ran Yanovich

**Affiliations:** 1 Heller Institute of Medical Research, Sheba Medical Center, Tel Hashomer, ISRAEL; 2 Ariel University Center of Samaria, Ariel, ISRAEL; 3 Division of Medicine, Department of Gastroenterology, Hebrew University-Hadassah Medical Center, Jerusalem, ISRAEL; 4 The, Orthopaedic Department, Assaf Harofeh Medical Center, Zerifin and The Sackler School of Medicine, Tel Aviv University, Zerifin, Tel Aviv, ISRAEL; 5 Military Performance Division, U.S. Army Research Institute of Environmental Medicine, Natick, MA, USA

**Keywords:** Basic training, Bone, Calcium, Vitamin D, Nutrition

## Abstract

**Background:**

Appropriate and sufficient dietary intake is one of the main requirements for maintaining fitness and health. Inadequate energy intake may have a negative impact on physical performance which may result in injuries among physically active populations. The purpose of this research was to evaluate a possible relationship between dietary intake and stress fracture occurrence among combat recruits during basic training (BT).

**Methods:**

Data was collected from 74 combat recruits (18.2 ± 0.6 yrs) in the Israeli Defense Forces. Data analyses included changes in anthropometric measures, dietary intake, blood iron and calcium levels. Measurements were taken on entry to 4-month BT and at the end of BT. The occurrence of stress reaction injury was followed prospectively during the entire 6-month training period.

**Results:**

Twelve recruits were diagnosed with stress fracture in the tibia or femur (SF group). Sixty two recruits completed BT without stress fractures (NSF). Calcium and vitamin D intakes reported on induction day were lower in the SF group compared to the NSF group-38.9% for calcium (589 ± 92 and 964 ± 373 mg·d^-1^, respectively, *p *< 0.001), and-25.1% for vitamin D (117.9 ± 34.3 and 157.4 ± 93.3 IU·d^-1^, respectively, *p *< 0.001). During BT calcium and vitamin D intake continued to be at the same low values for the SF group but decreased for the NSF group and no significant differences were found between these two groups.

**Conclusions:**

The development of stress fractures in young recruits during combat BT was associated with dietary deficiency before induction and during BT of mainly vitamin D and calcium. For the purpose of intervention, the fact that the main deficiency is before induction will need special consideration.

## Background

Military personnel represent a unique population exposed to intense physical and cognitive demands during both training and operational missions. Typically, military service commences with basic training (BT) which is characterized by intense physical training, emotional and mental stress [1]. It should be emphasized that such a challenging environment is enhanced during combat recruit training.

Individuals seeking to enhance physical performance through participation in arduous physical activity, particularly athletes and combat soldiers, must adhere to an appropriate and sufficient dietary intake [2,3]. Inadequate energy intake can prolong recovery from illness and injury, depress immune function, and have a negative impact on physical performance in both training and operational activities [4,5]. It has also been reported that inadequate energy intake, especially restrictive dietary patterns, may be associated with the development of stress fractures [4].

The U.S. Army has published regulations which define the nutritional responsibilities of the Surgeon General of the Army, the Navy, and the Air Force. These regulations, referred to as the Military Dietary Reference Intakes (MDRI), evaluate the effects of environmental factors on energy and nutrient requirements and outline nutrition education policy [5]. The MDRI is a quantitative estimate of the recommended dietary intake for healthy military populations based on US national standards [5]. The Nutritional Standards for Operational and Restricted Rations (NSOR) was established to take into account the higher energy expenditure in field exercises and other operational and logistic factors relevant for training [5]. As an example, studies that quantified energy expenditure during military operations report that Special Forces soldiers had up to 45% higher absolute energy expenditure compared to their non-combat counterparts [6,7]. During prolonged training periods, if energy deficits occur, this may endanger the general health of the soldiers and reduce the muscle mass and bone strength needed for optimal performance. Of note, previous reports have found an association between insufficient dietary intake and increased risk for stress fractures among military recruits [8-10].

Bone overuse injuries, also referred to as stress reactions and stress fractures, are the most common overuse injuries among combat soldiers and are observed most frequently among young army recruits who undergo strenuous exercise during basic training [11]. The occurrence of severe cases of stress fracture has even reached rates as high as 64% in the Finnish army [12] and 31% in the Israeli Defense Forces (IDF) [13].

Stress fractures have been found to be related to several risk factors, both intrinsic and extrinsic [14], over most of which we have no control [13]. These include bone geometry parameters (studied thoroughly in the IDF), gender and hormonal factors, and genetic predisposition. Studies on bone density have been contradictory [14], and biochemical markers of bone turnover are also probably not related to stress fractures [15]. Calcium deficiency has been found deterrent to bone quality in animal models [16,17] but studies on athletes and soldiers have been less conclusive. Calcium and vitamin D are probably important in women [18] and in Finnish males (who may be effected by the latitude) [19], but in general, there is not enough data on males. Lappe et al managed to reduce stress fracture incidence in female navy recruits by about 20% [9]. Smoking (present or history) has also been found to be related to stress fractures, particularly in the US [20], and is possibly related to risk taking behavioral patterns. However, this finding has not been reproduced consistently in other militaries [19,21].

The purpose of this study was to evaluate nutritional intake in male combat recruits before induction and during a 4-month BT period. A secondary aim was to determine the possible association between specific nutrient intake, serum markers of nutrition and the occurrence of bone stress reactions.

## Methods

### Subjects

The study sample consisted of 74 combat male recruits from an elite combat unit in the IDF. Volunteers were recruited at the beginning of a mandatory three year military service program. All volunteers had successfully completed a strenuous 4-day sorting course 3 months prior to their induction. The military training protocol was designed to prepare the soldiers for combat missions, and included general physical fitness, physical work under pressure, hand to hand combat training, direct fire battle, leadership development and stressful combat conditions.

The study was approved by both Institutional Review Board Committees of the IDF and Sheba Medical Center, and the U.S. Army Research Institute of Environmental Medicine at Natick, MA, and all the participants signed informed consent for participation in the study.

### Data collection

Data on the soldiers' anthropometric measurements, nutritional habits, iron indices, and serum calcium were collected on induction and again after 4 months. Blood samples were also taken 6 months after induction. A medical evaluation was conducted at baseline and then bi-weekly during the 6-month period by two orthopedic surgeons in order to detect the presence of stress fracture and other overuse injuries.

### Anthropometric measurements

Anthropometric measurements included body weight, height, body fat percentage and calculation of body mass index (BMI). Height (cm) was measured using a stadiometer (± 1 cm) and body weight (kg) was determined with a metric scale (± 100 gr). In order to avoid errors, the same researcher completed all anthropometric measurements at each data collection point. Body fat percentage was calculated according to Siri from a 4-site skin fold thickness (biceps, triceps, subscapula, and suprailiac) [22] using Lange skin fold calipers (Beta Technology, Santa Cruz, CA).

### Nutritional assessment

Food intake was assessed using the food frequency questionnaire (FFQ), developed and validated for the Israeli population by The S. Daniel Abraham International Center for Health and Nutrition at the Ben-Gurion University, Israel [23,24]. It is a long-term dietary assessment tool consisting of 126 food items divided into nine food groups that can be analyzed for nutrient and food group intake, such as: 1) eggs, milk, and milk products; 2) fats (including sauces); 3) chicken, meat, and fish; 4) bread and baked products; 5) starches and legumes; 6) fruit; 7) vegetables; 8) snacks and cookies; and 9) beverages. Subjects completed the FFQ with the assistance of a dietitian at two time points: on induction, referring specifically to the previous 6 months, and then again 4 months after starting BT, referring to the 4 months of BT. All food input was to be reported [25]. The FFQ analysis produces daily nutritional intake of proteins, carbohydrates, total fat, iron, folate, vitamins (D, B_6_, and B_12_), calcium, zinc, and magnesium. Our study referred to the MDRI and NSOR and we compared both raw data and data normalized for body weight.

### Hematology and blood chemistry

Blood samples representing nutritional status were collected at three time points (0, 4, 6 months) and the following components were analyzed: hemoglobin (Hgb), iron, transferrin, ferritin, folic acid, vitamin B_12_, and calcium. Approximately 30 cc's of venous blood samples were obtained by antecubital venipuncture into tubes (BD Vacutainer; Becton, Dickinson and Company^®^, 2002 BD) containing the appropriate anticoagulant. All samples were taken during the morning hours (0600-0700 h), in a sitting position after an overnight fast (6-10 h) and no exercise. The samples were placed in ice and sent within four hours to be processed and analyzed at the Sheba Medical Center Laboratories (Hematology and Biochemistry). Blood counts were performed on fresh blood using an automated analyzer (Cell-Dyn^® ^3000; Abbott Diagnostics, Abbott Park, IL) for measuring Hgb values. Serum ferritin was measured using an electrochemiluminescence immunoassay (Roche Elecsys^®^, Roche Diagnostics GmbH, Mannheim, Germany, reference: of 16-293 ng/ml). Serum iron was measured with a commercial kit on Olympus (AU2700, reference ranges 60-170 μg/dL). Vitamin B_12 _and folic acid levels were determined with an automated analyzer (Architect Abbott Diagnostics). Serum transferrin was measured with an immunoturbidimetric assay (Tina-quant^® ^with Roche Diagnostics GmbH, Mannheim, Germany, reference ranges 193-378 mg/dL). Transferrin saturation was calculated according to the following formula: transferrin saturation (%) = serum iron/serum transferrin. Blood calcium was measured using a commercial kit on Olympus (AU2700, reference values: 8.5-10.9 mg/dl). Radioimmunoassay (RIA) was used to measure 25(OH)D levels (DiaSorin, Stillwater, MN, reference range 30.0-74.0 ng/ml). Parathyroid hormone (PTH) was measured by immunoassay with chemiluminescent detection on the Immulite 2000 (Diagnostics Products Corporation, Los Angeles, CA, reference range 12.0-72.0 ng/L).

Hematological deficiencies were established as follows: anemia was diagnosed at Hgb levels of less than 14 g/dl, and ferritin levels (< 20 ng/ml).

### Nutrition provided to recruits

The recruits had 3 main meals and 3 snacks a day. Breakfast (7-8 am, about 2 h after awakening), which included porridge, bread, 1-2 eggs, cream cheese, vegetables, olives, jam and additional savory spread for the bread (avocado, chickpea etc, depending on the season). A chocolate drink (200 ml milk) and milk desserts were also served. Dinner (12-1 pm) included soup, a meat/chicken/fish portion (200 grams) 2 salads and a cooked vegetable, a starch (potatoes/rice/macaroni), bread and a fruit desert. Supper (5-6 pm) included a main portion (1-2 eggs/macaroni etc) bread, cream cheese and spreads, hard cheese, and all other components served at breakfast. The three intermediate snacks were usually 1-2 sandwiches with jam or chocolate spread. All food was provided at no cost to the recruits.

Dietary supplements were not given or encouraged, though they were not prohibited and their use was not monitored. Formally, recruits were allowed to get additional snacks at the canteen, but they were not given access to the canteen on a regular basis. They might also have eaten extra food sent by relatives.

### Injury assessment

Injury surveillance and bone stress injury diagnosis took place over the course of the entire 6-month training period. We used three sources of data: the unit physicians treating the recruits recorded overuse injuries separately in a personal surveillance table. Two orthopedic surgeons examined the recruits every 2-3 weeks and registered their findings in the recruits' central army Computerized Patient Record (CPR). Stress reactions and fractures were diagnosed by clinical examination and confirmed by radiography or bone scintigraphy [26]. Sixty two recruits without clinical signs of stress reactions and those whose imaging ruled out a stress reaction or fracture were classified as the NSF group. Twelve recruits with stress fractures of the tibia or femur confirmed by imaging were classified as the SF group. Since the mechanism for developing stress fractures in the metatarsals is fatigue and not remodeling, as in the long bones [27], we focused only on stress fractures of long bones.

### Statistical analysis

Data analysis was performed using the Statistical Package for the Social Sciences software version 15.1 (SPSS INC., Chicago, IL). Comparisons between study groups over the time points, and at each phase were performed using repeated measures ANOVA (groups and time; α < 0.05) followed by pairwise comparisons using Student's t-test with adjustments for multiple comparisons by Tukey-Kramer. Analysis of the nutritional data produced descriptive statistics including mean, standard deviation, standard error, and range.

## Results

Out of the seventy four recruits who completed all data collection during the 6-month training program, twelve recruits were diagnosed with stress fractures of the long bones (tibia and femur) by imaging during the 6-months. The results of the measured variables (i.e., anthropometry, nutritional consumption, and hematology) are presented for a total of 74 soldiers: 12 SF recruits vs. the 62 NSF recruits.

### Anthropometric measurements

On induction, body weight was not significantly different between the SF and the NSF groups (68.1 ± 4.5 and 71.5 ± 6.8 kg, respectively) but the two groups' body weight did differ significantly (*p *< 0.05) at the end of BT (68.6 ± 4.7 and 72.6 ± 6.2 kg, respectively). No significant statistical differences were evident among the rest of the anthropometric measurements (height, body fat percentage, BMI) between the two study groups.

### Nutritional consumption

On induction the SF group reported lower nutritional intake of most nutrients compared to the NSF group with significant differences (*p *< 0.05) in carbohydrates (272 ± 104 and 369 ± 165 g, respectively), calcium (589 ± 92 and 964 ± 373 mg·d^-1^, respectively), and vitamin D (117.9 ± 34.3 and 157.4 ± 93.3 IU·d^-1^, respectively), as depicted in Table 1. Notably, carbohydrates, calcium, and vitamin D intakes for the SF group were 55.1%, 67.9%, and 59.6% less than the NSOR requirements, respectively. Normalized nutrient intake (for body weight) was also significantly different (*p *< 0.05) between the SF group and the NSF group for these three nutrients: Carbohydrates (4.00 ± 0.04 and 5.2 ± 0.04 g·kg^-1^, respectively), calcium (8.6 ± 0.04 and 13.5 ± 0.02 mg·d^-1^·kg^-1^, respectively), and vitamin D (1.73 ± 0.13 and 2.2 ± 0.07 IU·d^-1^·kg^-1^, respectively).

**Table 1:** The Study groups' daily nutritional intake (mean ± SD) at induction and after 4-months basic training (BT)in relation (%) to the Nutritional Standards for Operational and Restricted Rations (NSOR) requirements

	NSF (N = 62)		SF (N = 12)	
	**Induction**	**End BT**	**Induction**	**End BT**

**Energy **(kcal)	2824 ± 1086 (78.4%)	2587 ± 879 (71.9%)	2325 ± 974 (64.6%)	2447 ± 879 (68.0%)

**Proteins **(g)	128.6 ± 62.8 (141%)	114.0 ± 42.4 (125%)	111.7 ± 43.1 (123%)	131.7 ± 48.3 (145%)

**Carbohydrates (**g)	369 ± 165* (74.7%)	335 ± 178 (67.8%)	272 ± 104 (55.1%)	285 ± 129 (57.7%)

**Total Fat (**g)	100.3 ± 40.5 (32.0%)	89.7 ± 31.5 (31.2%)	84.5 ± 14.8 (34.5%)	108.0 ± 35.0 (34.4%)

**Iron **(mg)	18.0 ± 7.7^#^ (120%)	15.2 ± 5.5 (101%)	16.1 ± 5.1 (107%)	14.6 ± 4.8 (97.3%)

**Folate **(μg DFE)	448 ± 198^#^ (112%)	364 ± 132 (91.0%)	362 ± 108 (90.5%)	332 ± 126 (83.0%)

**Vitamin D **(IU)	157.4 ± 93.3*^#^ (78.7%)	119.2 ± 53.1 (59.6%)	117.9 ± 34.3 (59.0%)	121.6 ± 46.1 (60.8%)

**Vitamin B_6 _**(mg)	3.0 ± 1.3^#^ (231%)	2.3 ± 0.8 (177%)	2.8 ± 1.1 (215%)	2.3 ± 0.9 (177%)

**Vitamin B_12 _**(μg)	7.1 ± 4.0^#^ (296%)	4.8 ± 2.3 (200%)	5.9 ± 3.2 (246%)	6.2 ± 3.0 (258%)

**Calcium **(mg)	964 ± 373*^#^ (96.4%)	679 ± 236 (67.9%)	589 ± 92 (58.9%)	609 ± 171 (60.9%)

**Zinc **(mg)	15.8 ± 6.6^# ^ (105%)	12.5 ± 4.3 (83.3%)	14.7 ± 4.6 (98.0%)	12.4 ± 2.6 (82.9%)

**Magnesium **(mg)	394 ± 155^# ^ (93.8%)	338 ± 118 (80.5%)	320 ± 129 (76.2%)	318 ± 108 (75.7%)

* *p *< 0.05 NSF vs. SF at the same phase

^# ^*p *< 0.05 for the same group at different phases

Dietary intakes for the NSF group decreased significantly (*p *< 0.05) during BT from pre-induction values for almost all measured variables: carbohydrates by 15.6%, folate by 18.8%, vitamin D by 24.3%, calcium by 29.6%, zinc by 20.9%, and magnesium by 14.2%. No significant changes occurred in any of the measured variables among the SF group.

During BT, the recruits' nutritional intake (both groups) did not meet the NSOR recommendations for total energy and most nutrients, including carbohydrates, total fat, folate, vitamin D, calcium, zinc, and magnesium. Vitamin B_12_, B_6 _and protein values, however, were greater than the NSOR recommendations, both pre and post BT as depicted in Table 1.

### Blood biochemistry variables

Throughout the study, all subjects from the 2 groups demonstrated mean hematological values in accordance with normal levels for young, healthy males. Nevertheless, mean values of Hgb, folic acid, serum calcium, iron, ferritin and transferrin saturation decreased significantly (*p *< 0.05) during BT for both groups as depicted in Table 2. After 6 months, Hgb, serum calcium, ferritin and transferrin saturation remained lower, whereas folic acid and iron levels increased.

**Table 2:** Biochemical and biomarker variables (mean ± SD) at induction (0), after 4-month BT (4), and after 6 months from induction (6)

	NSF (N = 62)			SF (N = 12)		
**Month**	**0**	**4**	**6**	**0**	**4**	**6**

**HGB (g/dl)**	15.7 ± 0.9^+Δ^	14.2 ± 0.9	14.2 ± 0.9	15.6 ± 0.5^+Δ^	14.6 ± 0.8	13.9 ± 1.0

**Folic acid serum (ng/dl)**	6.1 ± 2.6^+Δ^	3.9 ± 1.7	7.1 ± 2.5	7.1 ± 3.7^+^	3.8 ± 1.9	7.0 ± 2.4

**Calcium total (mg/dl)**	10.1 ± 0.4^+Δ^	9.7 ± 0.4	9.8 ± 0.3*	9.9 ± 0.3^Δ^	9.6 ± 0.4	9.5 ± 0.2

**Iron (μg/dl)**	118.9 ± 51.4^+^	65.4 ± 24.1	130.4 ± 71.5*	121.2 ± 53.8^+^	66.8 ± 22.6	71.7 ± 27.2

**Transferrin (mg/dl)**	303.9 ± 48.2	306.0 ± 28.5	307.6 ± 41.4	264.0 ± 53.5	302.4 ± 67.5	295.9 ± 50.4

**Ferritin (ng/ml)**	54.3 ± 30.0^+Δ^	42.6 ± 22.5	22.8 ± 9.6	57.4 ± 30.2^Δ^	38.7 ± 19.0	31.9 ± 16.5

**Transferrin saturation (%)**	39.1 ± 12.7^+^	21.4 ± 8.5	23.4 ± 9.2	41.1 ± 13.5^+^	22.1 ± 11.7	24.2 ± 10.8

**25(OH)D (nmol/L)**	75.3 ± 16.3	64.6 ± 10.2	72.4 ± 13.8	70.5 ± 16.5	63.0 ± 12.4	66.4 ± 16.4

**PTH (ng/L)**	32.4 ± 14.9	50.2 ± 17.1	32.1 ± 19.9	31.9 ± 18.5	43.8 ± 17.8	37.4 ± 22.7

* *p *< 0.05 NSF vs. SF at the same examination date

^+ ^*p *< 0.05 at the same group, between induction and end of BT

^Δ ^*p *< 0.05 at the same group, between induction and 6-month

On induction and after 4-months BT no differences were observed in all of the measured variables (Hgb, folic acid, calcium, iron, transferrin, ferritin, 25(OH)D and PTH) between the SF and the NSF groups. However, significant differences (*p *< 0.05) were found after 6 months in serum calcium (9.5 ± 0.2 and 9.8 ± 0.3 mg·dl^-1^, respectively) and iron (71.7 ± 27.2 and 130.4 ± 71.5 μg·dl^-1^, respectively).

## Discussion

The aim of this study was to evaluate a possible relationship between nutritional intake before induction and during BT and long bone stress fracture occurrence among male combat recruits. We monitored 74 recruits through a 6-month period (4 months BT and 2 months advanced training) of intense physical and mental training. This period is also characterized by a major change in nutritional habits, partially resulting from eating in mess and rations provided in the field. One of the consequences of these changes in lifestyle and training regime was that 16% of the recruits developed stress fractures in their long bones, similar to previous reports on recruits performing this type of training.

Stress fracture susceptibility is multi-factorial. A main factor in the mechanism of long bone stress fractures is imbalance in osteoclasis and osteogenesis that are over-activated to enable the bone remodeling necessary for the bone strengthening [28-31]. In this milieu of excessive osteogenic needs, it is reasonable to assume that dietary deficiency of calcium and/or vitamin D could contribute to stress fractures, as has previously been implicated in rickets, osteomalacia, osteopenia, osteoporosis, fractures and other cases of excessive bone resorption [32].

In this group of recruits, we found considerable dietary deficiency. First, despite the high energy needs during this period of training, the recruits consumed only 70% of the energy recommendations of the NSOR, with the NSF group reporting an 8.4% decrease in their BT total energy intake compared with the pre-induction total energy intake. This low intake may be explained by the presence of fundamental stressors in the military environment, such as periodic food restrictions, sleep deprivation, mental burden, and constant physical evaluations. These findings are in accordance with previous studies pointing to the fact that military personnel normally consume insufficient energy, whether or not they are provided with an adequate amount of food [33]. In this study, the deficient energy intake was not associated with a weight loss but rather an increase of body weight during BT by 1.5%. This is also in line with previous studies, specifically that in this training program the gained weight was in lean body mass and not in fat [34].

We are concerned that our participants did not meet MDRI requirements. These deficiencies were observed for nearly every nutrient evaluated in the FFQ. The highest deficiencies were for vitamin D and calcium in the SF group, both around 60% of the MDRI before induction and also during BT. Of note, among the NSF group, vitamin D intake was the second most deficient variable, reported to be consumed at a level of 78.7% from MDRI before induction and at even a lower level of 59.6% during BT. In our study, the SF recruits reported 41.0% less initial calcium and vitamin D intakes on induction day than the MDRI recommendations. Although vitamin D3 (cholecalciferol) is either formed in the skin after exposure to sunlight or obtained from nutritional sources, especially fatty fish [32], most IDF soldiers use sunscreen and wear long-sleeved clothing during military training. This may limit vitamin D3 synthesis, and therefore, the importance of balanced nutritional intake, especially of vitamin D and calcium, should be emphasized, even though we did not actually find low serum levels of vitamin D.

Release of PTH is controlled by the level of calcium in the blood, with low blood calcium levels causing an increase in PTH. The main purpose of this hormone is calcium homeostasis. It is therefore not surprising that in these healthy young recruits, we did not find any pathological PTH or calcium values. A slight trend towards higher levels of PTH in the 4-month BT may represent a lack of dietary calcium. However PTH differences between SF and NSF or between induction values and 4 or 6 month values were not significant.

Furthermore, we found that the SF group compared to NSF had insufficient intake of zinc and magnesium (93.8% to 80.5% and 98.0% to 82.9% of the MDRI, respectively) throughout BT. In addition to calcium, minerals and trace elements such as zinc and magnesium are involved in skeletal growth and are required for normal bone metabolism. An adequate intake of these dietary components is therefore necessary to assure optimal bone quality and prevention of bone loss [35].

It is also evident that during BT, SF soldiers developed iron deficiency and anemia symptoms associated with 39% low transferrin saturation (< 16%), 36.4% ferritin deficiency (< 20 ng/ml), and 37.9% hemoglobin deficiency (< 14 g/dl). Notably, similar findings were observed in previous studies involving elite Israeli male athletes [36,37], and in female combatants [38]. Moreover, it is important to note that iron and ferritin levels are a part of an innovative prediction model for stress fractures in young female recruits during basic training, which managed to correctly predict stress fracture occurrence in 76.5% of a sample population [39].

The study has several limitations. Assessing food consumption based on a person's memory is always problematic. This is more so with recruits in a very intense physical and mental training schedule. We also did not monitor personal initiatives in taking nutritional supplements. Previous surveys have demonstrated this to be negligible, with recruits showing minimal interest in calcium and vitamin D. Another problem is the lack of finding of low vitamin D levels, despite the dietary deficiency. We also did not measure serum zinc levels, however, following these results it would seem beneficial to measure these levels for future research on recruits.

## Conclusions

The main conclusion from this study is that, contrary to previous beliefs, male infantry recruits in the IDF are nutritionally deficient, specifically in calcium and vitamin D, and those who were more deficient developed more stress fractures. This directly arouses the debate on supplying supplements, following Lappe et al. in the US Navy female recruits [9]. But it is doubtful whether such an intervention is justified for a 20% decrease in stress fracture incidence in the IDF, and further research would be necessary to prove the efficacy in IDF male combat recruits. Another issue is related to the fact that there was dietary deficiency before induction, making intervention by the military at the most appropriated time more complicated. Based on our findings it might be plausible to perform nutritional screening (e.g., questionnaire) of elite combat recruits on induction and possibly assess the deficient subjects for serum levels. We could then treat those with low levels.

It should therefore be emphasized that while engaging in strenuous physical training, proper nutrient intake may act as a long-term protector against bone resorption and stress fracture development, and is recommended for maintaining healthy bones [40].

## Competing interests

The authors declare that they have no competing interests.

## Authors' contributions

DSM and RY conceived the study idea and analysed the data. DSM, YA, RKE, and RY designed the study. YA and RY carried out data collection. ASF conducted the orthopaedic examinations. DSM and RY drafted the manuscript. All authors contributed to the interpretation of results, critically reviewed the manuscript for intellectual content, and gave approval of the final version of the manuscript to be published.
